# Supplementary figures and images for: Global variation of low bone mineral density in special olympics adult athletes with intellectual and developmental disability—A cross-sectional study
Source: PLOS Glob Public Health. 2025 Oct 7;5(10):e0005125. doi: 10.1371/journal.pgph.0005125 (PMC12503286; doi:10.1371/journal.pgph.0005125)

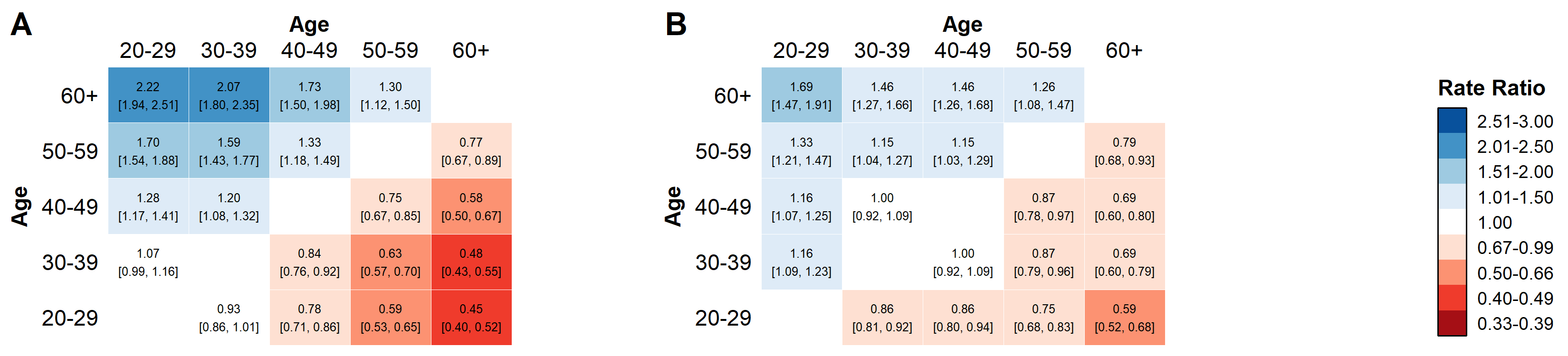

Supplement: S1 Fig — Each grid contains the prevalence rate ratio and [95% CI]. Colors gradients correlate with the magnitude of the prevalence rate ratios; note all 95% CIs that contain one have their tiles colored white. (TIF) [file pgph.0005125.s001.tif]

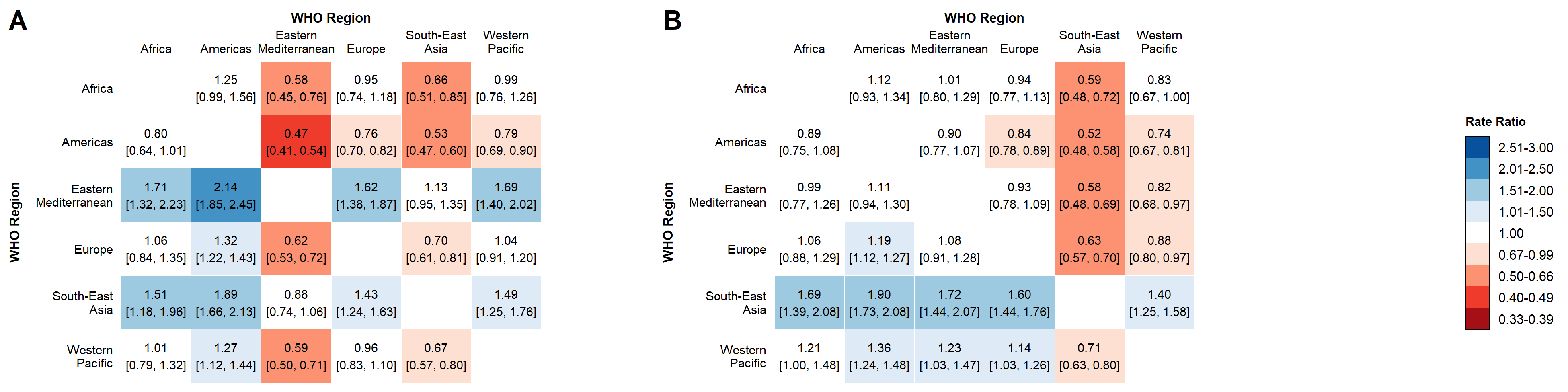

Supplement: S2 Fig — Each grid contains the prevalence rate ratio and [95% CI]. Colors gradients correlate with the magnitude of the prevalence rate ratios; note all 95% CIs that contain one have their tiles colored white. (TIF) [file pgph.0005125.s002.tif]

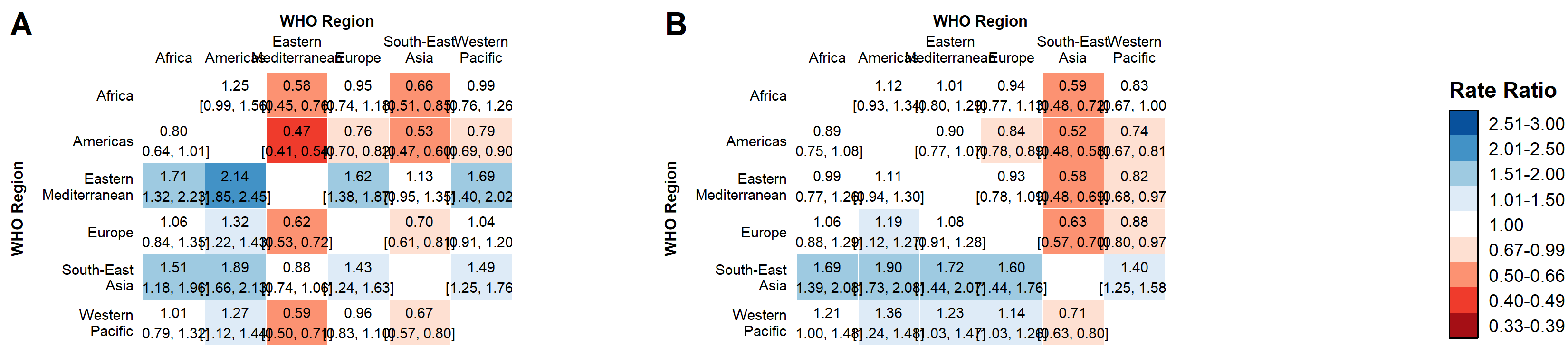

Supplement: S3 Fig — Each grid contains the prevalence rate ratio and [95% CI]. Colors gradients correlate with the magnitude of the prevalence rate ratios; note all 95% CIs that contain one have their tiles colored white. (TIF) [file pgph.0005125.s003.tif]
