# Supplementary material for: Global variation of low bone mineral density in special olympics adult athletes with intellectual and developmental disability—A cross-sectional study
Source: PLOS Glob Public Health. 2025 Oct 7;5(10):e0005125. doi: 10.1371/journal.pgph.0005125 (PMC12503286; doi:10.1371/journal.pgph.0005125)
Supplement: S1 Table — The age distributions for each WHO region were normalized to the WHO Americas age distribution. See S2 Fig to compare the prevalence rate ratios of LBMD between WHO regions within a given sex. (DOCX) [file pgph.0005125.s004.docx]

**S1 Table.** Prevalence rates, with 95% confidence intervals, of LBMD for females and males. The age distributions for each WHO region were normalized to the WHO Americas age distribution. See S2 Fig to compare the prevalence rate ratios of LBMD between WHO regions within a given sex. ^†^

| **WHO Region** | **Total** | **Female** | **Male** |  |
| --- | --- | --- | --- | --- |
| **Africa** | 29.5%   (25.3% - 33.6%) | 30.3%   (23.5% - 37.0%) | 28.9%   (23.7% - 34.1%) |  |
| **Americas** | 24.9%   (24.3% - 25.5%) | 24.0%   (23.1% - 25.0%) | 25.6%   (24.7% - 26.4%) |  |
| **Eastern**   **Mediterranean** | 38.3%   (34.3% - 42.3%) | 51.6%   (44.7% - 58.6%) | 28.3%   (23.7% - 32.8%) |  |
| **Europe** | 31.1%   (29.7% - 32.4%) | 31.8%   (29.7% - 34.0%) | 30.5%   (28.8% - 32.2%) |  |
| **Southeast**   **Asia** | 47.3%   (44.0% - 50.5%) | 45.4%   (40.1% - 50.7%) | 48.6%   (44.5% - 52.7%) |  |
| **Western**   **Pacific** | 33.0%   (30.7% - 35.2%) | 30.6%   (26.9% - 34.3%) | 34.8%   (31.9% - 37.6%) |  |

^†^ All data are derived from Special Olympics athletes ≥20 years old in the Special Olympics Healthy Athletes database.
