# Supplementary material for: Global variation of low bone mineral density in special olympics adult athletes with intellectual and developmental disability—A cross-sectional study
Source: PLOS Glob Public Health. 2025 Oct 7;5(10):e0005125. doi: 10.1371/journal.pgph.0005125 (PMC12503286; doi:10.1371/journal.pgph.0005125)
Supplement: S2 Table — In addition, the Chi-square statistic and associated p-value are presented for each comparison. (DOCX) [file pgph.0005125.s005.docx]

**S2 Table.** Prevalence rates and prevalence rate ratios, with 95% confidence intervals, of LBMD where within each region the female age distribution is normalized to the male age distribution. In addition, the Chi-square statistic and associated p-value are presented for each comparison. ^†^

| **WHO Region** | **Female** | **Male** | **Female vs Male** | **χ^2^** | ***p*-value** |
| --- | --- | --- | --- | --- | --- |
| **Africa** | 21.0%  (15.0% - 27.0%) | 20.8%  (16.1% - 25.4%) | 1.01  [0.70, 1.45] | 0.00 | 1.0000 |
| **Americas** | 23.8%  (22.8% - 24.8%) | 25.6%  (24.7% - 26.4%) | 0.93  [0.88, 0.98] | 7.08 | 0.0078 |
| **Eastern**  **Mediterranean** | 43.8%  (36.9% - 50.7%) | 28.3%  (23.8% - 32.8%) | 1.55  [1.24, 1.94] | 13.52 | 0.0002 |
| **Europe** | 31.2%  (29.0% - 33.3%) | 30.2%  (28.5% - 31.9%) | 1.03  [0.94, 1.13] | 0.45 | 0.5036 |
| **Southeast**  **Asia** | 45.3%  (40.0% - 50.6%) | 39.9%  (35.8% - 43.9%) | 1.14  [0.97, 1.33] | 2.44 | 0.1180 |
| **Western**  **Pacific** | 26.3%  (22.8% - 29.8%) | 30.8%  (28.1% - 33.6%) | 0.85  [0.73, 1.00] | 3.54 | 0.0597 |

^†^ All data are derived from Special Olympics athletes ≥20 years old in the Special Olympics Healthy Athletes database.
