# Supplementary material for: Global variation of low bone mineral density in special olympics adult athletes with intellectual and developmental disability—A cross-sectional study
Source: PLOS Glob Public Health. 2025 Oct 7;5(10):e0005125. doi: 10.1371/journal.pgph.0005125 (PMC12503286; doi:10.1371/journal.pgph.0005125)
Supplement: S3 Table — See S3 Fig to compare the prevalence rate ratios of LBMD between WHO regions within a given sex. (DOCX) [file pgph.0005125.s006.docx]

**S3 Table.** Prevalence rates, with 95% confidence intervals, of failing to achieve PBM for 20–29-year-olds by sex and WHO region. See S3 Fig to compare the prevalence rate ratios of LBMD between WHO regions within a given sex. ^†^

| **WHO Region** | **Total** | **Female** | **Male** |  |
| --- | --- | --- | --- | --- |
| **Africa** | 45.8%  (40.7% - 51.0%) | 48.6%  (40.2% - 56.9%) | 43.9%  (37.4% - 50.5%) |  |
| **Americas** | 48.0%  (46.9% - 49.1%) | 45.3%  (43.6% - 47.0%) | 49.9%  (48.5% - 51.4%) |  |
| **Eastern**  **Mediterranean** | 62.3%  (57.6% - 67.0%) | 66.9%  (59.2% - 74.6%) | 59.1%  (53.2% - 65.0%) |  |
| **Europe** | 59.0%  (57.0% - 61.0%) | 57.6%  (54.4% - 60.9%) | 59.9%  (57.3% - 62.5%) |  |
| **Southeast**  **Asia** | 66.7%  (63.3% - 70.1%) | 75.9%  (70.9% - 80.8%) | 60.3%  (55.8% - 64.8%) |  |
| **Western**  **Pacific** | 55.6%  (52.7% - 58.5%) | 54.1%  (49.2% - 59.1%) | 56.6%  (53.1% - 60.2%) |  |

^†^ All data are derived from Special Olympics athletes ≥20 years old in the Special Olympics Healthy Athletes database.
