# Supplementary material for: Global variation of low bone mineral density in special olympics adult athletes with intellectual and developmental disability—A cross-sectional study
Source: PLOS Glob Public Health. 2025 Oct 7;5(10):e0005125. doi: 10.1371/journal.pgph.0005125 (PMC12503286; doi:10.1371/journal.pgph.0005125)
Supplement: Attachment — Submitted filename: Feedback_PGPH-D-24-03053.pdf [file pgph.0005125.s009.pdf]

Feedback

Abstract

The statistical details (e.g., Z-scores, p-values, confidence intervals) are quite dense and might overwhelm a general reader. Simplify or selectively report statistics in the abstract so instead of the full results say something like 'LBMD prevalence increased significantly with age in both males and females' leave the details for the results section in the full paper unless they are central to the key message. Also, consider using person-first language (e.g., "adults with IDD" instead of "individuals with IDD") consistently for tone and inclusivity.

Introduction

A very comprehensive introduction, however the reader goes through several paragraphs before reaching the aim so you could bring the main aim of the study forward earlier. The overall flow could be improved by briefly stating the knowledge gap also a bit earlier – after introducing the increased risk of LBMD and fractures insert a linking sentence like 'Despite the risks, population level data on BMD status of people with IDD remains sparse….This study aims to address that gap by….' This helps orientate the reader earlier and enhances coherence. The definition of PBM ("failure to accrue optimal PBM (T-score of ≥ 0.0)") might be confusing, usually PBM refers to the maximal bone mass, not necessarily defined by a T-score. There is a heavy density of statistics perhaps summarise some of the data and statistics more succinctly to maintain flow, this will maintain the impact while improving accessibility without overwhelming the reader or diluting the narrative.

Methods

This methods section is thorough and includes the appropriate necessary components. It outlines clearly the data source, the ethics approvals, the tools and classification criteria and a comprehensive summary of the statistical analysis plan. One aspect that is important to acknowledge explicitly is the potential variability between the devices used, confirm that appropriate calibrations or adjustments were made to ensure comparison. Also note your limitations that whilst these devices are convenient, they are not diagnostic and DXA is the only gold standard recognised therefore I would suggest wen mentioning t-scores you identify that they are QUS t-scores and be consistent throughout your article.

Results

Good consistency between text content and tables and figures. Would suggest label clarity in text versus table – table uses WHO regions (Africa, Americas, etc) whereas text references 'normalised to the Americas region' consider adding a brief note in methods or results to explain normalisation. Add a footnote to the table clarifying WHO regions classifications, that all data are adults age ≥20yrs and that percentage in brackets equals percent within regional total or overall group.

"Southeast Asia females having the highest rates (75.9%) followed by Southeast Asia females" (line 185-186). This looks like a typo or repetition - did you mean another group followed? Needs clarity.

Discussion

A comprehensive discussion is presented with the findings consistent with the tables etc in the results section. Key concepts are highlighted clearly, and the public health relevance is emphasised with solid citation and actionable recommendations. Some ideas are repeated maybe unnecessarily for example - Lines 201–203 and 262–264 both state that LBMD and PBM failure vary by age, gender, and region. And the section 221–230 revisits screening guidelines after PBM failure has already been discussed. These could be condensed where possible for stronger impact. Line 217–219: *"Cultural and religious practices..."* – consider softening language to avoid unintended cultural generalizations. Perhaps: "...may contribute to lower sun exposure and vitamin D levels, potentially affecting BMD." Your conclusion has strong content but could be slightly tightened/ sharpened for impact

Overall, this is a well-written and timely article that addresses an important and underexplored area of health among individuals with intellectual and developmental disabilities. The authors are to be commended for their comprehensive analysis and thoughtful discussion, well done.
