# Supplementary material for: Global variation of low bone mineral density in special olympics adult athletes with intellectual and developmental disability—A cross-sectional study
Source: PLOS Glob Public Health. 2025 Oct 7;5(10):e0005125. doi: 10.1371/journal.pgph.0005125 (PMC12503286; doi:10.1371/journal.pgph.0005125)
Supplement: Attachment — Submitted filename: Review Summary Bone Health.docx [file pgph.0005125.s008.docx]

**Review summary of the article titled “ Bone Health Surveillance in Adults with Intellectual and Developmental Disability(IDD)”**

This research is relevant and significant as it assesses the prevalence of low bone mineral density (LBMD) in a high risk vulnerable population of adults with intellectual and developmental disability. The authors defined their population of interest and had included sufficient evidence within their introduction to explain their rationale for conducting the study. The authors reported the knowledge and dearth in research that exists on LBMD in this population, justifying their need to conduct the research.

**Title**:

The title of the study is clear but should include that it was conducted among special olympic athletes as it is a special subgroup within the population of adults with IDD.

**Abstract:**

This was structured and well written. The methodology should include that BMD testing was conducted by licensed clinicians and Institutional review board approval was obtained. Statistical analysis method employed and the statistical package used can also be included in one sentence.

**Methodology.**

The authors included most of the relevant information required for this section. It would have been useful to include a brief background on how the secondary data was originally collected.

It would also be useful to know if the data had more sociodemographic or clinical information that would have been relevant to the data analysis.

**Results**

The authors had included a description table on the socio demographic profile of the study population.The statistical methods employed were expected for type of study design and the number of variables available within the dataset

**Discussion section**:

This section was well structured and key implications of the results were highlighted. The authors should evaluate their research findings against more studies conducted in populations with IDD or special olympic population with IDD if available. The evaluation should explore and highlight the settings within the other studies that may have led to the similarities or differences with their study findings. The authors should also include potential areas for future research based on their research as this was not clear in the discussion section.

**References**

The manuscript was well referenced.

**Evaluation and recommendation**

Overall, this was research done among a specialized sub-group of high risk individuals for LBMD. This is critical research of publishable status.It is a well written manuscript requiring minor changes.
