# Supplementary material for: Global variation of low bone mineral density in special olympics adult athletes with intellectual and developmental disability—A cross-sectional study
Source: PLOS Glob Public Health. 2025 Oct 7;5(10):e0005125. doi: 10.1371/journal.pgph.0005125 (PMC12503286; doi:10.1371/journal.pgph.0005125)
Supplement: Attachment — Submitted filename: Article suggestions.docx [file pgph.0005125.s010.docx]

Respected Editor,

Thank You for the opportunity to review the article. Please find my comments below –

**Title** – Kindly include “Athletes with IDD” to give clarity on the population of interest

**Methods -**

- Is there a possibility of duplication of data of participants who appeared more than once to the Olympics? If yes, which values were included and excluded?
- Are BMD T scores comparable across geographical regions? Was cross region adjustments and standardization done before interpretation? Kindly add reference
- Sensitivity and specificity of the Quantitative USG used? Kindly add reference
- Can WHO classification of osteoporosis thresholds apply to QUS results? Kindly add reference
- Implications of restricting assessment only to a single site (heel)
- Were appropriate confounders adjusted for? Kindly mention the variables adjusted for in analysis.
- Scope of adding more relevant variables in the regression model – physical activity, BMI, nutrition, medication history, etc

**Results**

- Add atleast the supplementary table 1 and 2 (containing prevalence of LBMD) in the main article after combining them in the same table. As this is your primary objective, these results should feature in the main article
- Can we make meaningful comparison of different regions when the disparity in sample sizes is significant?

**Discussion**

- Need to mention the implications of all possible selection and measurement bias in detail in discussion
