# Supplementary material for: Global variation of low bone mineral density in special olympics adult athletes with intellectual and developmental disability—A cross-sectional study
Source: PLOS Glob Public Health. 2025 Oct 7;5(10):e0005125. doi: 10.1371/journal.pgph.0005125 (PMC12503286; doi:10.1371/journal.pgph.0005125)
Supplement: Attachment — Submitted filename: Response_to_Reviewers.docx [file pgph.0005125.s012.docx]

Response to Reviewers

# General

**Reviewer Editor Comment:** We do not publish any copyright or trademark symbols that usually accompany proprietary names, eg (R), (C), or TM (e.g. next to drug or reagent names). Please remove all instances of trademark/copyright symbols throughout the text, including ® on pages 2, 4, 9, 11 and 12.

**Response:** removed copyright symbols and recommended

# Title

**Reviewer 3 Comment:** Kindly include “Athletes with IDD” to give clarity on the population of interest

**Response:** We have now changed the title to "Global Variation of Low Bone Mineral Density in Special Olympics Adult Athletes with Intellectual and Developmental Disability " in accordance with the reviewers comments.

**Reviewer C Comment:** The title of the study is clear but should include that it was conducted among special olympic athletes as it is a special subgroup within the population of adults with IDD.

**Response:** We have now changed the title to "Global Variation of Low Bone Mineral Density in Special Olympics Adult Athletes with Intellectual and Developmental Disability " in accordance with the reviewers comments.

**Reviewer Editor Comment:** Please include the study design in the title, as suggested by STROBE. (<https://www.strobe-statement.org/>)

**Response:** We have now changed the title to "Global Variation of Low Bone Mineral Density in Special Olympics Adult Athletes with Intellectual and Developmental Disability " in accordance with the reviewers comments.

# Abstract

**Reviewer B Comment:** The statistical details (e.g., Z-scores, p-values, confidence intervals) are quite dense and might overwhelm a general reader. Simplify or selectively report statistics in the abstract so instead of the full results say something like ‘LBMD prevalence increased significantly with age in both males and females’ leave the details for the results section in the full paper unless they are central to the key message.

**Response:** As per the reviewer's suggestion we have removed 95% confidence intervals, z-scores, and p-values from the abstract to open the abstract to make it more accessible to the general reader.

**Reviewer C Comment:** Statistical analysis method employed and the statistical package used can also be included in one sentence.

**Response:** As per the reviewer's suggestion we have added the following sentence to the abstract.
"Chi-square, rate ratios with 95% confidence intervals, and binomial logistic regression were calculated in R version 4.3.1 to analyze LBMD and PBM across age, WHO regions, and sex. "

**Reviewer B Comment:** Also, consider using person-first language (e.g., “adults with IDD” instead of “individuals with IDD”) consistently for tone and inclusivity.

**Response:** We have updated the wording throughout the manuscript in order to be more inclusive as per the reviewer's suggestion.

**Reviewer C Comment:** This was structured and well written.

**Response:** We thank the reviewer for the positive feedback.

**Reviewer C Comment:** The methodology should include that BMD testing was conducted by licensed clinicians and Institutional review board approval was obtained.

**Response:** We have added the following sentence to the abstract methodology per the reviewer's suggestion.
"BMD testing was conducted by licensed clinicians and institutional review board approval was obtained to use this deidentified data."

# Introduction

**Reviewer B Comment:** A very comprehensive introduction, however the reader goes through several paragraphs before reaching the aim so you could bring the main aim of the study forward earlier. The overall flow could be improved by briefly stating the knowledge gap also a bit earlier – after introducing the increased risk of LBMD and fractures insert a linking sentence like ‘Despite the risks, population level data on BMD status of people with IDD remains sparse….This study aims to address that gap by….’ This helps orientate the reader earlier and enhances coherence.

**Response:** We thank the reviewer and have added the following text to the introduction:
"Despite well-documented risks, population-level data on BMD in people with intellectual and developmental disabilities (IDD) remain limited. This study aims to address this gap by examining the trajectory of quantitative ultrasound (QUS) BMD T-scores from early adulthood onward, to determine when and how these scores begin to diverge from those observed in the general population."

**Reviewer B Comment:** The definition of PBM (“failure to accrue optimal PBM (T-score of ≥ 0.0)”) might be confusing, usually PBM refers to the maximal bone mass, not necessarily defined by a T-score.

**Response:** We agree that the wording may have been a little confusing and have thus changed the introduction slightly to read "In addition to genetics and certain medical conditions, low PBM or the failure to accrue a healthy PBM (QUS T-score of ≥ 0.0)"
As the reviewer is well aware, that PBM is unique to each individuals and is generally achieved before the age of 40. We wanted to highlight in this manuscript that many 20-29 year olds in this study already have a PBM < 0 and since BMD decreases with age they are closer to LBMD at their peak than is desired.

**Reviewer B Comment:** There is a heavy density of statistics perhaps summarise some of the data and statistics more succinctly to maintain flow, this will maintain the impact while improving accessibility without overwhelming the reader or diluting the narrative.

**Response:** As per the reviewers comments we have removed some of the data and statistics from the introduction.

# Methods

**Reviewer Editor Comment:** In line 89, it is mentioned that this is a secondary data study, and in line 114, you state “there were 25,868 athletes ≥ 20 years old from 179 countries who received BMD testing.” In Methods section, it is important to clarify the universe size (N) and the number of athletes in the universe who received BMD testing (n), so readers will understand how representative the sample is. Additionally, please explain possible reasons why some athletes were not included.

**Response:** We have now included how we cleaned the data. We have now clarified that only Special Olympic athletes whose sex (i.e., Male, Female) is not missing was used in this analysis.
"Between 2011-2023, there were 26,152 athlete screenings where athletes received BMD testing. Only athlete screenings where the athlete’s age was between 20 and 89 years old, had a biological sex of female or male, and had the country of the athlete were used in this analysis. Therefore, this study focuses on 25,868 athlete screenings and represents Special Olympics athletes ≥ 20 years old from 179 countries who received BMD testing."

**Reviewer Editor Comment:** The female-male variable is mentioned in the paper. However, it is not clear whether athletes of other genders were excluded from the analysis, not accepted in the competitions, or if you decided to use biological sex for the analysis or to exclude other genders. Please clarify this in the methods. This should be clarified

**Reviewer 3 Comment:** Is there a possibility of duplication of data of participants who appeared more than once to the Olympics? If yes, which values were included and excluded?

**Response:** There is the possibility that the same athlete was screened multiple times. However, it would be highly unlikely for them to be screened multiple times for the same age. Thus, even if the same athlete were screened, each age of data is a unique data point. We have changed the demographics in the results section to clarify that the data is based on athlete screenings and not athletes screened. Quality Control (QC) procedures, including routine calibration as outlined in the manufacturer’s operator manuals and volunteer training protocols, were followed daily prior to conducting BMD testing with athletes.
"Between 2011-2023, there were 26,152 athlete screenings where athletes received BMD testing. Only athlete screenings where the athlete’s age was between 20 and 89 years old, had a biological sex of female or male, and had the country of the athlete were used in this analysis. Therefore, this study focuses on 25,868 athlete screenings and represents Special Olympics athletes ≥ 20 years old from 179 countries who received BMD testing. "

**Reviewer 3 Comment:** Are BMD T scores comparable across geographical regions? Was cross region adjustments and standardization done before interpretation? Kindly add reference

**Response:** Based on other comments we added the following citation to the methods section.
International Society for Clinical Densitometry. Position Development Conference on Quantitative Ultrasound (QUS) in Bone Densitometry: Guidelines for Clinical Application, Quality Control, and Training. [Internet]. 2019. Available from: https://www.iscd.org
While we cannot find an explicit citation stating that BMD scores need to take into account regional differences before comparing, we believe that a lack of such citations infers that the devices do not need to be adjusted to match unique regional physiological conditions when recording T scores. Without a regional adjustment and given that BMD are using WHO categories, we feel that our WHO regional comparisons are appropriate since we age normalized the WHO regions to give each region the same age distribution when we compared LBMD across regions.

**Reviewer B Comment:** One aspect that is important to acknowledge explicitly is the potential variability between the devices used, confirm that appropriate calibrations or adjustments were made to ensure comparison.

**Response:** We added and adjusted the methods section to address the reviewer's concern:
"Special Olympics Health Screenings are performed by clinical volunteers who follow Special Olympics guidelines. Quality Control (QC) procedures, including routine calibration as outlined in the manufacturer’s operator manuals and volunteer training protocols, were followed daily prior to conducting BMD testing with athletes, to ensure the delivery of reliable, accurate, and consistent results. These practices included daily calibration with phantoms, image quality checks, reproducibility testing, regular maintenance, and adherence to regulatory standards."

**Reviewer 3 Comment:** Sensitivity and specificity of the Quantitative USG used? Kindly add reference

**Response:** Added descrition of sensitivity and specificity of QUS as recommended incluidng references. "QUS showed a pooled sensitivity of ~80% and specificity of ~65% when using T-score cutoffs comparable to DXA and is effective at identifying individuals at high risk of low bone density (22,23)"

**Reviewer 3 Comment:** Can WHO classification of osteoporosis thresholds apply to QUS results? Kindly add reference

**Response:** While QUS are not the gold standard, they are highly correlated to DXA scores which are the gold standard and whose sensitivity and specificity is now included in the methods section. While we acknowledge that DXA would be ideal, QUS scores allows Special Olympics to reach a larger population and refer them onto further care and/or better diagnostic tests such as DXA. QUS has unique benefits including absense of radiation exposure, the equipment is more affordable, portable, easy to train individuals to operate. It provides immediate results that don't have to be interpreted by radiologists, and are more readily available in low-resource areas and situations. The testing process is well accepted by people with IDD due to simplicity of measurement and opportunity to converse and interact easily with the operator during procedures. People in wheel chairs can be tested without several people having to lift them onto complicated equipment in a clinical enviroment. Matschiner E, Serban O, Fodor D, Blaivas M, Horn R, Koch J, Jakobi ML, Grevelding L, Osterwalder J, Srivastava D, Dietrich CF. Ultrasound in bone fracture diagnosis - a comparative meta-analysis and systematic review. Med Ultrason. 2025 Mar 2;27(1):52-62. doi: 10.11152/mu-4407. Epub 2024 Jun 28. PMID: 39078997

**Reviewer 3 Comment:** Implications of restricting assessment only to a single site (heel)

**Response:** We have added to the manuscript the following citation to address this concern:
International Society for Clinical Densitometry. Position Development Conference on Quantitative Ultrasound (QUS) in Bone Densitometry: Guidelines for Clinical Application, Quality Control, and Training. [Internet]. 2019. Available from: https://www.iscd.org
Thus, while QUS is not the gold standard, a single heel site measurement is appropriate for this methodology.

**Reviewer 3 Comment:** Were appropriate confounders adjusted for? Kindly mention the variables adjusted for in analysis.

**Response:** We have updated the wording throughout the manuscript in order to be more inclusive as per the reviewer's suggestion.

**Reviewer 3 Comment:** Scope of adding more relevant variables in the regression model – physical activity, BMI, nutrition, medication history, etc

**Response:** As per another reviewer's comment we added WHO region to the binomial regression models. We were limited in the variables that we could add to the regression model based on the data we had available. Also, we felt that other confounding variables (e.g., nutrition) could add too much unwanted noise since BMD is scores are based on longer timeframe than variables such as current nutritional habits in addition to issues of the reliability of self-report data.

**Reviewer C Comment:** It would also be useful to know if the data had more sociodemographic or clinical information that would have been relevant to the data analysis.

**Response:** We have added this to the the limitations of the study:
"In addition, since longitudinal data does not exist for the Special Olympics athletes in this study, we were not able to account for confounding factors that impact BMD over time such as nutrition, activity levels and medication use. "

**Reviewer B Comment:** This methods section is thorough and includes the appropriate necessary components. It outlines clearly the data source, the ethics approvals, the tools and classification criteria and a comprehensive summary of the statistical analysis plan.

**Response:** We thank the reviewer for the positive feedback.

**Reviewer 4 Comment:** please comment upon the reliability of using QUS for BMD screening. How does this compare to DEXA so that we know how much to accept these results?

**Response:** We have added more material to the Methods section to address the reviewers concern.
"QUS showed a pooled sensitivity of ~80% and specificity of ~65% when using T-score cutoffs comparable to DXA and is effective at identifying individuals at high risk of low bone density (22,23)."

**Reviewer B Comment:** Also note your limitations that whilst these devices are convenient, they are not diagnostic and DXA is the only gold standard recognised therefore I would suggest wen mentioning t-scores you identify that they are QUS t-scores and be consistent throughout your article.

**Response:** We have adjusted the manuscript to clearly state that all T-Scores are QUS T-Scores throughout the manuscript.

**Reviewer C Comment:** The authors included most of the relevant information required for this section.

**Response:** We thank the reviewer for the positive feedback.

**Reviewer C Comment:** It would have been useful to include a brief background on how the secondary data was originally collected.

**Response:** We have now added to the methodology to discuss how the data is collected:
"BMD testing was conducted at HA screenings by licensed clinicians or clinicians-in-training. Quality control procedures were part of clinician training provided prior to BMD testing with athletes (18,19). BMD QUS T-scores were obtained using the Hologic Sahara Bone Densitometer, the Osteosys Sonost 3000, or the GE Achilles Express 3000. These ultrasound devices measure broadband ultrasound attenuation (BUA) and speed of sound (SOS) to determine QUS T-scores."
"Between 2011-2023, there were 26,152 athlete screenings where athletes received BMD testing. Only athlete screenings where the athlete’s age was between 20 and 89 years old, had a biological sex of female or male, and had the country of the athlete were used in this analysis. Therefore, this study focuses on 25,868 athlete screenings and represents Special Olympics athletes from 179 countries who received BMD testing."

# Results

**Reviewer 4 Comment:** At several points, there were comments upon the increasing % of risk of LBMD per year of age. Did the study team actually plot out the rates by year of age or by decile of age? If they did assess each year of age individually, it would be really nice to see that data in the supplemental figures.

**Response:** We did build a binomial logistic regression model based ages 20 to 89, treating age as a continuous variable. We have the plotted the model with the raw data, which we will share with you here, but do not plan on adding the plot as a supplemental plot since we are not convinced that they have value.
Figure X. Binomial logistic regression models where the black line represents the model, the dots represent the data the model was built on. Red dots are Special Olympic athletes with LBMD and blue dots are athletes that do not have LBMD. A) Female crude binomial logistic regression model, B) Male crude binomial logistic regression model, C) Female adjusted binomial logistic regression model , and D) Male adjusted binomial logistic regression model.

**Reviewer 3 Comment:** Add at least the supplementary table 1 and 2 (containing prevalence of LBMD) in the main article after combining them in the same table. As this is your primary objective, these results should feature in the main article

**Response:** As suggested by the reviewer, Supplemental Table 1, which was combined with Supplemental Table 2 are now Table 2 in the main text of the manuscript.

**Reviewer 3 Comment:** Can we make meaningful comparison of different regions when the disparity in sample sizes is significant?

**Response:** We appreciate the reviewer’s concern regarding the disparity in sample sizes across regions. The statistical methods employed—Chi-square tests, rate ratios, and logistic regression—are designed to account for differences in group sizes. Nonetheless, we acknowledge that estimates from smaller groups (e.g., n=465) may be less stable, and we have interpreted those results with appropriate caution, including reporting confidence intervals to reflect this uncertainty.

**Reviewer B Comment:** Good consistency between text content and tables and figures. Would suggest label clarity in text versus table – table uses WHO regions (Africa, Americas, etc) whereas text references ‘normalised to the Americas region’ consider adding a brief note in methods or results to explain normalisation.

**Response:** For all LBMD analysis we normalized the data so that the rates could be more fairly compared. When comparing across regions an sexes, we normalize to match the age distribution of the Americas region. When comparing the rates LBMD of females and males within a region, we normalize the females to match the distribution of the males. We have now added more details regarding normalization in the methods section.
"When comparing LBMD rates across WHO regions, results were age-normalized by gender to the Americas region, so that each region would have the same age structure. When comparing LBMD prevalence rates within WHO regions, females were age-normalized to match the age distribution of the males in their region. In order to age normalize the data, ages were binned in ten-year bins. "

**Reviewer B Comment:** Add a footnote to the table clarifying WHO regions classifications, that all data are adults age >20yrs and that percentage in brackets equals percent within regional total or overall group.

**Response:** We have added footnotes to the data tables as per your request and that of another reviewer.
"All data are derived from Special Olympics athletes ≥20 years old in the Special Olympics Healthy Athletes database."

**Reviewer B Comment:** "Southeast Asia females having the highest rates (75.9%) followed by Southeast Asia females" (line 185-186). This looks like a typo or repetition - did you mean another group followed? Needs clarity.

**Response:** Yes, thank you for pointing out this error. Below is the updated results:
" There is geographic variation in failing to achieve PBM with Southeast Asia females having the highest rates (75.9% (70.9 - 80.8%)) followed by Southeast AsiaEastern Mediterranean females (66.9% (59.2 – 74.6%))"

**Reviewer Editor Comment:** Line 109: Binomial logistic regression is mentioned. It appears to be a crude binomial logistic regression with age as a continuous variable, even though region and the female-male variable are confounders in this relationship. I suggest running both crude and adjusted logistic regression in this case.

**Response:** We had already separated the analysis for males and females and have now adjusted the wording to mention this in the methods. We also added wording and methods to create adjusted models that take into account WHO regions. Below is the new wording:
"Both crude and adjusted bBinomial logistic regressions wereas performed for females and males to measure the increase in LBMD with age as a continuous variable and WHO region as a confounding variable. "
We added the adjusted model to the results section.
"The crude binomial logistic regression model revealed that for every year of age, the prevalence of LBMD increased 1.43% [1.11%, 1.76%] (Z = 8.70, p <.0001) for males and 2.50% [2.12%, 2.89%] (Z = 12.83, p <.0001) for females. When adjusting the binomial logistic regression models for WHO regions, the prevalence of LBMD increased 1.78% [1.45%, 2.11%] (Z = 10.46, p <.0001) for males and 3.13% [2.74%, 3.53%] (Z = 15.37, p <.0001) for females for every year of age."

**Reviewer Editor Comment:** Lines 139–140: Age was managed as a string variable. Please clarify in the methods section why two types of age analysis were used, or consider unifying them.

**Response:** We have added more description to the methods describing the practice of binning ages in order to normalize the data.
"In order to age normalize the data, ages were binned in ten-year bins."

**Reviewer Editor Comment:** Line 116: There is a reference not inserted.

**Response:** I am not sure what the reviewer is referring to. There is a reference to Table 1 on line 116. I am not sure what else we should or could reference here.

**Reviewer Editor Comment:** Table 1: Please include p-values for the observed differences in these variables.

**Response:** We added a Pearson Chi-Square test on the age distribution and WHO regions produces the following statistics: X-squared = 1278.4, df = 20, p-value < 0.0001 and comparing the sex distribution and WHO regions produces the following statistics: X-squared = 71.825, df = 5, p-value < 0.0001.

**Reviewer Editor Comment:** General: Add the source of each table and figure as a footnote.

**Response:** As per the reviewer's suggestion we have now added footnotes to every table explaining where the data comes from.

**Reviewer Editor Comment:** Line 138: Please change “rate ratio” to “prevalence rate ratio” in this line and elsewhere in the text where “prevalence” is missing (e.g., line 169).

**Response:** We have made the suggested changes.

**Reviewer Editor Comment:** Line 152: I suggest reviewing this text. The prevalence rate of LBMD (the outcome) is being presented, not the prevalence rates of males and females.

**Response:** We have changed the writing as recommended:
"The prevalence rate of LMBD for females and males with LBMD across the WHO regions normalized to the Americas age distribution. Error bars represent 95% confidence intervals."

**Reviewer Editor Comment:** Line 188: Figure 4 was attached as Figure 3. Please review this.

**Response:** We apologize for this oversight.

**Reviewer Editor Comment:** Supplementary Figure 1: The heat map of prevalence rate ratio shows redundant information when comparing the risk of LBMD (e.g., comparing 20–29 years to 60–69 years, then 60–69 years to 20–29 years). I suggest revising this figure to exclude redundant information and, for example, compare 60–69 years males prevalence rate ratios to 60–69 years females prevalence rate ratios, and so on for other categories.

**Response:** We have added more of a description to the figure captions to explain the rows are compared to the correspinding column. While we acknowledge that upper right triangle of the figure is the inverse of the lower left triangle we feel that there is merit in including both triangles so as to more easily compare one row to all other columns. While Supplemental Figure 1 does not have as much variation due to the ordinal nature of age bins, Supplemental Figures 2 and 3 have nominal data. Take for instance Supplemental Figure 2B. It is much easier to see that South East Asian males have higher rates of LBMD than every other region by simply looking across the row and seeing everything is blue. If we were to do as suggested, then South East Asian males would have four blue boxes and one red associated with it or 4 red and 1 blue which visually is not as straightforward a story. That being said we respectfully decline to change the figures, but will add more information to the figure caption to better guide the reader.

**Reviewer Editor Comment:** Supplementary Figure 2: The same comment as for Supplementary Figure 1 applies.

**Response:** See above.

**Reviewer Editor Comment:** Supplementary Figure 3: The same comment as for Supplementary Figure 1 applies.

**Response:** See above.

**Reviewer Editor Comment:** Supplemental Table 4: This table should be sorted as follows: second column—Total, Male, Female, Male vs Female Ratio, p-value (from Table 2). Do not use scientific notation for p-values (e.g., 2.99 × 10⁻²); use standard notation (e.g., 0.0290, four decimals). This suggestion applies to all tables and figures in the article. For p-values less than 0.0001, note as <0.0001.

**Response:** We have changed all p-values to be reported to 4 decimal places as are the requirements of this journal.

**Reviewer Editor Comment:** Supplemental Table 2: With the previous changes, this table is not needed.

**Response:** After combining Supplemental Tables 1 and 2 we have now removed the original Supplemental Table 2.

**Reviewer Editor Comment:** Supplemental Table 3: This table should be sorted as follows: second column—Total, Male, Female, Male vs Female Ratio, p-value. Review the title; it refers to the prevalence of LBMD (the outcome), not the prevalence of females or males.

**Response:** We have changed the Figure caption as per the reviewer's suggestion. We have also moved the Total column to the second column. However, we are not adding p-values since they do not fit in the structure of this table. The data in this table is meant to compare the rates of LBMD across WHO regions within a single sex. Supplemental Figure 2 is used to show the statistical differences in prevelance rates of LBMD between WHO regions for a specific sex.

**Reviewer Editor Comment:** Supplemental Table 4: This table should be sorted as follows: second column—Total, Male, Female, Male vs Female Ratio, p-value (from Table 5).

**Response:** We have combined Supplemental Tables 4 and 5 as the reviewer suggested. We are not going to add a total % column because 1) the total appears in previous supplemental table and 2) the female percentage with LBMD is normalized to the male making the total by region confusing to interpret.

**Reviewer Editor Comment:** Supplemental Table 5: With the previous changes, this table is not needed.

**Response:** Has been combined with the original Supplemental Table 4.

**Reviewer Editor Comment:** Supplemental Table 6: This table should be sorted as follows: second column—Total, Male, Female, Male vs Female Ratio, p-value.

**Response:** We have moved the total column as per the reviewer's suggestion. We are not adding the p-value and rate ratio from Supplemental Table 5 since that statistic does not belong in this table. Instead we calculated prevalence rate ratios with 95% confidence intervals to compare the rates of PBM < 0.0 across the WHO Regions by sex. We have added wording to direct the reader to Supplemental Figure 3 to compare the prevalence rate ratios by gender across regions.

**Reviewer C Comment:** The authors had included a description table on the socio demographic profile of the study population.The statistical methods employed were expected for type of study design and the number of variables available within the dataset.

**Response:** We thank the reviewer for the positive feedback.

**Reviewer Editor Comment:** “Figure_2.tif, Figure_4.tif, Supplemental_Figure_2.tif and Supplemental_Figure_3.tif” files are over our file size limit of 10MB. This limit is in place for the convenience of reviewers, editors and readers. Please adapt this file so that the file size is below 10MB. You may find it helpful to consult our guidelines on compressing figures here: https://journals.plos.org/globalpublichealth/s/figures

**Response:** We have now compressed the figure files to be within the file size limit.

# Discussion

**Reviewer 3 Comment:** Need to mention the implications of all possible selection and measurement bias in detail in discussion

**Response:** We have included more information to address selection bias: This study has several limitations, including potential selection and measurement bias.” (Line 528). And, "Between 2011-2023, there were 26,152 athlete screenings where athletes received BMD testing. Only athlete screenings where the athlete’s age was between 20 and 89 years old, had a biological sex of female or male, and had the country of the athlete were used in this analysis. Therefore, this study focuses on 25,868 athlete screenings and represents Special Olympics athletes from 179 countries who received BMD testing."
We also added information to the methods to discuss how we limited measurement bias:
"Special Olympics Health Screenings are performed by clinical volunteers who follow Special Olympics guidelines. Quality Control (QC) procedures, including routine calibration as outlined in the manufacturer’s operator manuals and volunteer training protocols, were followed daily prior to conducting BMD testing with athletes, to ensure the delivery of reliable, accurate, and consistent results. These practices included daily calibration with phantoms, image quality checks, reproducibility testing, regular maintenance, and adherence to regulatory standards."

**Reviewer B Comment:** A comprehensive discussion is presented with the findings consistent with the tables etc in the results section. Key concepts are highlighted clearly, and the public health relevance is emphasised with solid citation and actionable recommendations.

**Response:** We appreciate the reviewer's positive comments.

**Reviewer B Comment:** Some ideas are repeated maybe unnecessarily for example - Lines 201–203 and 262–264 both state that LBMD and PBM failure vary by age, gender, and region. And the section 221–230 revisits screening guidelines after PBM failure has already been discussed. These could be condensed where possible for stronger impact.

**Response:** We appreciate the suggestion and have deleted lines 262-264 "This study underscores the high prevalence of LBMD among individuals with IDD and the critical need to address factors that influence bone health across age, gender, and geographic regions. " We also deleted "Over half of individuals with IDD in the 20-29-year-old age group" from lines 221-230 to reduce repeating information as suggested by the reviewer.

**Reviewer B Comment:** Line 217–219: "Cultural and religious practices..." – consider softening language to avoid unintended cultural generalizations.Perhaps: "...may contribute to lower sun exposure and vitamin D levels, potentially affecting BMD."

**Response:** Thank you for the recommendation. We have softened the language and changed the the text. It now reads "Certain lifestyle or environmental factors, such as limited sun exposure, may contribute to lower sun exposure, reduced vitamin D levels, potentially contributing to lower BMD in females from this region (36,37). "

**Reviewer B Comment:** Your conclusion has strong content but could be slightly tightened/ sharpened for impact

**Response:** To distinguish the conclusion from the rest of the manuscript, we have added a section header and tightened up the language. The conclusion now reads:
"This global study highlights the prevalence rates of LBMD for Special Olympics athletes with IDD across age-groups, sex, and WHO regions. The findings highlight regional disparities LBMD and PBM by WHO Global region, sex, and age. Preventive actions should begin in childhood and continue throughout life to optimize PBM, slow bone loss, and reduce the risk of fractures. Special Olympics plays a pivotal role in this space, offering opportunities for BMD screenings, physical activity, and health education that can positively impact BMD outcomes. Broader public health initiatives and policy changes are necessary to ensure people with IDD receive the support needed to develop and maintain healthy bones throughout their lives."

**Reviewer B Comment:** Overall, this is a well-written and timely article that addresses an important and underexplored area of health among individuals with intellectual and developmental disabilities. The authors are to be commended for their comprehensive analysis and thoughtful discussion, well done.

**Response:** We thank the reviewer for the positive feedback.

**Reviewer C Comment:** This section was well structured and key implications of the results were highlighted.

**Response:** We thank the reviewer for the positive feedback.

**Reviewer C Comment:** The authors should evaluate their research findings against more studies conducted in populations with IDD or special olympic population with IDD if available.

**Response:** We were not able to find any studies to directly compare to which is why we feel this work is important.
We do mention somewhat comparable studies in the discussion, but we know that is not exactly what the reviewer is looking for:
"People with IDD have higher rates of LBMD and fractures as well as earlier onset than the general population due to reduced mobility, lower physical activity, medication use, and nutritional deficiencies (14,27–29)."

**Reviewer C Comment:** The evaluation should explore and highlight the settings within the other studies that may have led to the similarities or differences with their study findings.

**Response:** Unfortunately, we were not able to find comparable studies where we could compare similarities and differences. We hope that this study encourages more research in this field.

**Reviewer C Comment:** The authors should also include potential areas for future research based on their research as this was not clear in the discussion section.

**Response:** We have now added future research that we feel will benefit this field:
"Future research should also explore public health initiatives and policy strategies to address modifiable risk factors common among people with IDD including food insecurity (46), poor nutrition (47,48), lack of routine BMD screenings (14,29,49), vitamin D testing (40), limited weight-bearing physical activity (50), hormone imbalances (9), and side effects of long-term medication use (51). Priority should be given to research into applying interventions that support the development of a healthy PBM to promote lifelong bone health in this population."

**Reviewer 1 Comment:** In the discussion section when comparing study findings with other research, it would be useful to include more details of the other research such as if the study was conducted in a similar population or among individuals with IDD.

**Response:** To our knowledge this is the first study of its kind to address LBMD in the Special Olympics athlete population, so each study we do compare to will be different. That said we have added information from other studies about LBMD in the IDD population:
"People with IDD have higher rates of LBMD and fractures as well as earlier onset than the general population due to reduced mobility, lower physical activity, medication use, and nutritional deficiencies (14,27–29). "

**Reviewer 4 Comment:** The authors did a nice job of introducing some initial ideas around LBMD prevention within this population. Do they have any specific recommendations for the younger populations with LBMD with regards to screening and prevention that we should perhaps include?

**Response:** First as we mention, participating in Special Olympics is one intervention that we recommend to people of all ages:
"Special Olympics is uniquely positioned to support bone health in people with IDD through both sport participation and health programming. As a global sports organization, Special Olympics provides year-round training and competition in weight-bearing sports that can help improve BMD. Additionally, many outdoor sports offer natural opportunities for vitamin D production through sun exposure. Beyond sports, the Special Olympics integrates BMD screenings and health education through HA. While HA screenings provide valuable information, not all athletes have access to them, underscoring the need for expanded educational and healthcare initiatives."
Also, while not directly addressing the reviewers concern we do recommend for future studies that could lead to interventions that could benefit younger populations:
"Future research should also explore public health initiatives and policy strategies to address modifiable risk factors common among people with IDD including food insecurity (46), poor nutrition (47,48), lack of routine BMD screenings (14,29,49), vitamin D testing (40), limited weight-bearing physical activity (50), hormone imbalances (9), and side effects of long-term medication use (51). Priority should be given to research into applying interventions that support the development of a healthy PBM to promote lifelong bone health in this population."

**Reviewer 5 Comment:** The recommendations for public health interventions are solid but could be improved by adding specific examples or strategies suited for low-resource settings.

**Response:** First Special Olympics is a global movement present in nearly even country including those with low-resources and even in low resource environments such as refugee camps. As we mention, participating in Special Olympics is one intervention that we recommend to people of all ages no matter where they are:
"Special Olympics is uniquely positioned to support bone health in people with IDD through both sport participation and health programming. As a global sports organization, Special Olympics provides year-round training and competition in weight-bearing sports that can help improve BMD. Additionally, many outdoor sports offer natural opportunities for vitamin D production through sun exposure. Beyond sports, the Special Olympics integrates BMD screenings and health education through HA. While HA screenings provide valuable information, not all athletes have access to them, underscoring the need for expanded educational and healthcare initiatives."
Second, we do not have specific recommendations but do agree on the importance of exploring startegies in low-resource settings, however, some of the recommendations we did add could apply to low-resource settings. Added this text to the Future Research section. "Future research should also explore public health initiatives and policy strategies to address modifiable risk factors common among people with IDD including food insecurity (46), poor nutrition (47,48), lack of routine BMD screenings (14,29,49), vitamin D testing (40), limited weight-bearing physical activity (50), hormone imbalances (9), and side effects of long-term medication use (51). Priority should be given to research into applying interventions that support the development of a healthy PBM to promote lifelong bone health in this population."

# References

**Reviewer C Comment:** The manuscript was well referenced.

**Response:** We thank the reviewer for the positive feedback.
